# Public Attitudes toward Gene Therapy in China

**DOI:** 10.1016/j.omtm.2017.05.008

**Published:** 2017-06-20

**Authors:** Jiang-Hui Wang, Rong Wang, Jia Hui Lee, Tiara W.U. Iao, Xiao Hu, Yu-Meng Wang, Lei-Lei Tu, Yi Mou, Wen-Li Zhu, Ai-Yong He, Shen-Yu Zhu, Di Cao, Lei Yang, Xiao-Bo Tan, Qing Zhang, Guan-Lu Liang, Shu-Min Tang, Ye-Di Zhou, Li-Jun Feng, Li-Jun Zhan, Nan-Nan Tian, Ming-Jie Tang, Ya-Ping Yang, Moeen Riaz, Peter van Wijngaarden, Gregory J. Dusting, Guei-Sheung Liu, Yan He

**Affiliations:** 1 Centre for Eye Research Australia, Royal Victorian Eye and Ear Hospital, East Melbourne, VIC 3002, Australia; 2 Ophthalmology, Department of Surgery, University of Melbourne, East Melbourne, VIC 3002, Australia; 3 College of Management, Shenzhen University, Shenzhen, Guangdong 518060, China; 4 Department of Ophthalmology and Visual Sciences, The Chinese University of Hong Kong, Hong Kong; 5 School of Marxism, Central South University, Changsha, Hunan 410011, China; 6 Department of Ophthalmology, The First Affiliated Hospital of Jinan University, Guangzhou, Guangdong 510630, China; 7 Department of Ultrasound, Guizhou Provincial People’s Hospital, Guiyang, Guizhou 550002, China; 8 Eye Hospital of China Academy of Chinese Medical Sciences, Beijing 100040, China; 9 Department of Orthopaedics, The Second Xiangya Hospital, Central South University, Changsha, Hunan 410011, China; 10 Department of Cardiothoracic Surgery, First Affiliated Hospital of Gannan Medical University, Ganzhou, Jiangxi 341000, China; 11 Department of Internal Medicine, Gaochun People’s Hospital, Nanjing, Jiangsu 211300, China; 12 Department of Ophthalmology, Affiliated Hospital of Chengde Medical University, Chengde, Hebei 067000, China; 13 Department of Ophthalmology, Zhejiang Hospital, Hangzhou, Zhejiang 310000, China; 14 Department of Ophthalmology, The Second Xiangya Hospital, Central South University, Changsha, Hunan 410011, China; 15 Department of Otorhinolaryngology, Head and Neck Surgery, Affiliated Xingtan Hospital of The First People’s Hospital of Shunde, Foshan, Guangdong 528313, China; 16 Department of Urology, The Central Hospital of Enshi Autonomous Prefecture, Enshi, Hubei 445000, China; 17 Department of General Surgery, Qinghai Provincial People’s Hospital, Xining, Qinghai 810000, China; 18 Department of Ophthalmology, Eye and ENT Hospital of Fudan University, Shanghai 200031, China; 19 Menzies Institute for Medical Research, University of Tasmania, Hobart, Tasmania 7000, Australia

## Main Text

Gene therapy, a medical procedure that delivers genetic materials into a person to potentially prevent or treat disease at its genetic roots, has long fascinated scientists, clinicians, and the general public. Although gene therapy has a checkered history, with several prominent adverse events in early trials, significant safety and efficacy improvements in the last two decades have catapulted the technology back to the center stage of medical research. As gene therapy rapidly progresses toward widespread clinical use, there is growing evidence of concern and skepticism in the scientific community and the general public alike.^1^ Among a range of concerns about gene therapy, balancing the benefits and risks of gene therapy stands out as a recurring theme. Ethical debate over the use of gene therapy for non-medical purposes, such as genetic enhancement of intelligence or physical aesthetics, has been robust. In addition, recurrent concerns have been raised about the ethical implications of gene therapy on human germlines.

Numerous studies of public attitudes to gene therapy or gene editing have been carried out in the past; however, these studies differ widely in their methods and the demographic and geographic attributes of the populations surveyed.^1^, ^2^ Of note, most of these studies have been conducted in Western countries. Given that China is the most populous nation in the world and has rapidly expanding capacity in gene therapy research, it is of utmost importance to understand the attitudes of the Chinese public and clinicians in relation to the application of gene therapy. Here we performed an online survey from 13,563 participants across China to explore attitudes toward gene therapy in different contexts (the complete questionnaire can be seen in Table S1). The majority of respondents completed all of the questions in the survey, giving a response rate of 97.3% (n = 13,201/13,563). Of valid respondents, 16.4% (n = 2,165/13,201) and 83.6% (n = 11,036/13,201) were clinicians (ascertained by self-report) and members of the general public, respectively. More than half of the respondents from both the clinician and general public groups were female (55.8% and 58.0%, respectively), and respondents’ ages ranged from 18 to 50 years. Other demographic information is provided in Table S2. We analyzed the geographic distributions of respondents of the two participant pools (the clinicians and the members of general public; Figure S1) and found that respondents from both groups were proportionally distributed across China based on the population distribution in each province.^3^

Several important findings have been obtained from our study. First of all, although gene therapy has been a familiar theme in the medical research community for decades, our study showed that both clinicians and members of the general public have much less awareness of gene therapy (63.1% and 29.9%, respectively) than genetically-modified (GM) food (90.2% and 83.4%, respectively; Q1 and Q2, Table S3 and Figure S2), a gene technology of which the public generally has a greater awareness and usually has been selected to compare with new technology previously.^2^ These findings are consistent with the results of other studies: Blendon et al.^1^ found that only 31% of the general public in the United States was familiar with gene therapy. The gap in awareness of gene therapy and GM food is likely related to the comparatively limited coverage of gene therapy in the media over the last few decades. In contrast to gene therapy, GM food is also perhaps more tangible to both clinicians and the general public because it is commonly encountered in supermarkets and the farming and containment of GM crops has gained media attention. These results may reflect that, while gene therapy is a prominent theme in the research community, it has not yet gained attraction with clinicians and medical students.

Here we also show that both clinicians and the general public mildly agreed that gene therapy would be beneficial for improving human health in the future, and clinicians responded more optimistically to this question than respondents from the general public (Q3, Table S3 and Figure S3). Interestingly, although both groups agreed on the promise of gene therapy as a future medical treatment, clinicians appeared to be more conservative than the public when asked if gene therapy would be a common therapy over the next few years (Q10, Table S3 and Figure S3). Moreover, both clinicians and the public strongly support the use of gene therapy to treat fatal or debilitating diseases in adults and fatal disease in children (Q5–Q8; Table S3 and Figure S3). Interestingly, we observed that respondents were more supportive of the use of gene therapy for fatal diseases than debilitating diseases, such as Alzheimer’s dementia and Parkinson’s disease. This is a trend that has also been described in other studies,^4^ and it may be attributed to the perception that gene therapy is not without significant risks and that, at this stage, the risk-benefit ratio of using gene therapy for medical conditions is perceived as inversely proportional to the severity of the disease.

In addition, there was substantially less support from both clinicians and members of the general public for the use of gene therapy for genetic enhancement for non-medical purposes, such as increasing intelligence and physical attributes (Q9, Table S3 and Figure S3). Results from previous studies support this finding.^1^, ^2^ Of note, we found that members of the general public were only neutral toward genetic enhancement, while clinicians disagreed with this application of gene therapy. Clinicians were less supportive of non-medical genetic enhancement, regardless of whether or not they had children. In contrast, members of the general public with children were more amenable to the idea than were those without (Table S4). The relative acceptance of genetic enhancement among members of the general public in China may reflect broader sociocultural pressures to excel in an increasingly competitive world.^5^ This finding draws attention to the need for political debate and legislative action to regulate the scope of non-medical genetic enhancement in China.

Indeed, these medical and ethical issues are the main concerns raised by clinicians and public respondents in our study (Q13, Table S3 and Figure S3). Our results showed that clinicians are more concerned about gene therapy going against nature (70.9%), followed by adverse medical side effects (68.9%), whereas the public respondents were primarily concerned about adverse medical side effects of gene therapy (72.0%), followed by high cost (61.9%). Interestingly, while both respondent groups were concerned about the safety of gene therapy, only clinicians were more concerned about gene therapy going against nature. This may mean that the public may not have as great an understanding as clinicians about the full implications and potential of gene therapy. Moreover, this may reflect the fact that the broader implications of gene therapy may not have been widely discussed in public due to a culture that does not always encourage freedom of debate on controversial subjects. Accordingly much of the debate about the ethics of gene therapy and editing in humans over the last three decades has come from commentators in Western countries.^1^, ^6^ Despite the concerns of clinicians about the “unnatural” nature of gene therapy, our results showed that both clinicians and the public were nearly neutral when asked if gene therapy will raise ethical issues (Q4, Table S3 and Figure S3). Indeed, the relatively loose regulation of the ethical review of clinical trials in China’s medical community means that gene therapy has been able to be more extensively researched in China compared to other countries. Although this is likely to have, in part, accelerated China’s position as a leading country in gene therapy research in humans compared to other countries with strict ethical regulations,^7^ we reflect that it is important to ensure that both clinicians and the general public in China are well-informed of the implications of gene therapy and that careful ethical consideration is given to this research to uphold both safety and core human rights.

Our study showed that attitudes and perceptions of gene therapy were influenced by specific demographic factors (see Tables S4 and S5). We found that women were significantly more likely than men to accept gene therapy for use in children with inherited diseases and in germline cells. This differs from the results of a study that showed men were found to be more accepting of all applications of gene editing compared to females.^2^ We also found that clinicians and public respondents with higher education or higher self-reported income were more likely to be supportive of the use of gene therapy for severe diseases. This result is consistent with a report that suggests people from developed countries with higher gross domestic product (GDP) per capita were more supportive of all health-related applications of gene editing.^2^ Respondents with self-reported religious affiliations were more likely to be significantly against using gene therapy to treat genetic diseases and were notably more reluctant to support government funding for gene therapy research. These results are perhaps not surprising, given that many religions have conservative positions in relation to other scientific developments, including human embryonic stem cell research. As expected, respondents with personal knowledge of an individual with a fatal debilitating or inherited disease were more accepting of the use of gene therapy for these conditions.

This study regarding the public perception of gene therapy was conducted via an online survey through social media, which can be used as a powerful tool to engage the public in biomedical research. However, there are several limitations to this study as a result of this method. First, there are many who do not have access to the internet or social media in China, and thus it is inevitable that our study has perhaps missed specific population groups, in particular, older individuals or those from regional or rural areas.^8^ Second, the use of the phrase “gene therapy” in the questions deals with non-therapeutic applications, such as genetic enhancement, which might potentially lead to an undesirable response because the “therapeutic tone” usually represents a positiveness. Third, a few participants in the general public group might have medical backgrounds that could have influenced their responses to the questions. Fourth, although we sampled respondents from different provinces across China in this survey, it may not absolutely reflect the Chinese population as a whole. For example, 84.4% of the public respondents in our study had at least a bachelor’s degree, which is a far higher proportion than in the general public,^9^ suggesting that there may be some recruitment bias. Of note, there were a few participants in the clinician group who did not have a college degree, suggesting that they were likely “barefoot doctors” who received basic medical training at county level to provide primary care to village populations.^10^ Follow-up studies using qualitative methods, such as focus groups and interviews, will be necessary to explore the attitudes of other stakeholders in greater depth.

In summary, our study is the first to investigate the attitudes of clinicians and members of the general public toward gene therapy in China. Our findings highlight the lack of knowledge of gene therapy among a large proportion of the public as well as around one-third of clinicians in China. Both groups were wary about using gene therapy for germline cells. However, the public was more amenable to genetic enhancement for non-medical reasons than clinicians. The safety of gene therapy was among the primary concerns for both the clinicians and public of China. Our results indicate that there is a need for both clinicians and the public to be more aware of the progress of gene therapy and its implications in order to keep the potential providers and receivers of this therapy well-informed. It also highlights the need for more ethical discussion regarding the uses of gene therapy from both China’s medical community and the general public to guide law and policy-making and the safe translation of gene therapy to the clinical setting.

## Conflicts of Interest

The authors declare no conflicts of interest.

## Supplemental Information

Document S1. Supplemental Materials and Methods, Figures S1–S3, and Tables S1–S5

Document S2. Article plus Supplemental Information
