# Supplementary material for: Public Attitudes toward Gene Therapy in China
Source: Mol Ther Methods Clin Dev. 2017 Jun 20;6:40–2. doi: 10.1016/j.omtm.2017.05.008 (PMC5480269; doi:10.1016/j.omtm.2017.05.008)
Supplement: Document S1. Supplemental Materials and Methods, Figures S1–S3, and Tables S1–S5 [file mmc1.pdf]

OMTM, Volume *6*

Supplemental Information

Public Attitudes toward Gene Therapy in China

Jiang-Hui Wang, Rong Wang, Jia Hui Lee, Tiara W.U. Iao, Xiao Hu, Yu-Meng Wang, Lei-Lei Tu, Yi Mou, Wen-Li Zhu, Ai-Yong He, Shen-Yu Zhu, Di Cao, Lei Yang, Xiao-Bo Tan, Qing Zhang, Guan-Lu Liang, Shu-Min Tang, Ye-Di Zhou, Li-Jun Feng, Li-Jun Zhan, Nan-Nan Tian, Ming-Jie Tang, Ya-Ping Yang, Moeen Riaz, Peter van Wijngaarden, Gregory J. Dusting, Guei-Sheung Liu, and Yan He

SUPPLEMENTAL MATERIALS

Ethical Review

This study was reviewed and approved by Human Subjects Research Ethics Board of the Second Xiangya Hospital, Central South University, China. All respondents gave informed consent prior to the survey and agreed to the use of their deidentified data for research purposes.

Study participants and survey dissemination

Clinicians and members of general public across China were invited to participate in a cross-sectional survey about attitudes to gene therapy. The survey was placed on Sojump platform (www.sojump.com) during the period from August 24 to November 2, 2016 to attract responses from members of the general population. The survey was promoted via the social media platform Wechat (https://wx.qq.com/) as well as other online tools such as Weibo China's Twitter equivalent through personal contacts, friends, and colleagues with the aim to balance out the young age bias given the social-media-based recruitment[1, 2].The participation of clinicians was achieved through survey dissemination in a variety of hospitals and medical conferences by trained research coordinators across the country and via Wechat. A brief introduction described gene therapy as well as the objectives of the survey and participants were offered an opportunity to opt out of the survey. The survey was closed at 3pm November 2, 2016 (GMT+8).

Survey instrument

In the development of our survey, we reviewed the literature, formulated questions, and conducted a cognitive phase testing in ten participants to refine the questionnaire [2-4]. Subsequently, changes were made to ensure questionnaire was understandable by both participant groups. The survey

was designed to gauge knowledge of genetically-modified (GM) food and gene therapy by using a five-point Likert scale (rating from 1 = never heard of it to 5 = know it very well) or agreement for questions in relation to gene therapy by using a seven-point Likert scale (rating from 1 = strongly disagree to 7 = strongly agree). The final structured survey (Table S1) was divided into eight sections: 1) a brief introduction to gene therapy, serving as background information; 2) whether the participant has heard of genetically modified food or gene therapy (Question 1 and 2); 3) general attitude to gene therapy and relevant ethical issues (Question 3, 4, and 10); 4) attitude to gene therapy if used to treat adults with fatal or debilitating diseases (Question 5 and 6); 5) attitude to gene therapy if used to treat children with fatal genetic diseases or to germline genetic modification or to enhance their genetic properties (Question 7, 8, and 9); 6) attitude to funding and legal support from the government for development of gene therapy in China (Question 11and 12); 7) main concerns about gene therapy applied in humans (Question 13); 8) demographics of participants including age, gender, residence, education, religion, children, occupation and financial condition (Question 14-24).

Data management and analysis

We analyzed the data by employing SPSS 20.0. Respondents rated all the questions on a five-point Likert scale or a seven-point Likert scale. To test the mean differences in participants' answers, we conducted t test or one-way ANOVA, while multiple linear regressions (Table S2) were conducted to examine the influences of demographic variables on the ratings. We considered the results to be statistically significant when p values were of <0 .05.

SUPPLEMENTAL TABLES

Table S1. A complete questionnaire is comprised of introduction.

A short introduction to gene therapy, provided for informational purposes

Gene therapy is a therapeutic strategy that corrects defects in the genetic material of a human being. The diseases can be prevented or treated by gene therapy via replace or modify a faulty gene of a patient's cells instead of using drugs or surgery. Gene therapy is currently only being used in clinic for the treatment of diseases that have no other cures. Gene therapy aims to eliminate the defect on the molecular level of the DNA via several approaches, including:

1. Replacing or correcting a mutated gene that causes disease with a healthy copy of the gene.
2. Inactivating, or "knocking out," a mutated gene that is functioning improperly.
3. Introducing a new gene into the body to help fight a disease.

Although gene therapy is a promising treatment option for a number of diseases (including some inherited and non-inherited disorders, a few types of cancer), the technique remains risky and is still under study to make sure that it will be safe and effective. Despite the rapid development and tremendous investment in health care and medical research in the last two decades, China is still behind the developed countries in terms of research and development of gene therapy.

The questionnaire below is to survey the attitude of the public and clinicians towards gene therapy in China. It will take you 10 minutes or so to finish it. Your participation in this project is entirely voluntary. If you do not wish to take part in, you are not obliged to. All comments and responses will be treated confidentially. The data will only be used for current and future analyses to address various research questions.

**Questions (Q)**		**Answer options**
1	Have you ever heard about genetically-modified food in the last 5 years?	Never heard of it, Head of it, Know a litter bit about it, Know a fair amount about it, Know it very well
2	Have you ever heard about gene therapy in the last 5 years?	Never heard of it, Head of it, Know a litter bit about it, Know a fair amount about it, Know it very well
3	Do you agree with that gene therapy will be helpful in addressing health needs of patients over the next few years?	Strongly disagree, Disagree, Mildly disagree, Neither agree nor disagree, Mildly agree,

		Agree, Strongly agree
4	Do you agree with that gene therapy poses significant ethical issues in terms of altering the human genome*?<br>*Human genome is the complete set of genetic material for human.	Strongly disagree, Disagree, Mildly disagree, Neither agree nor disagree, Mildly agree, Agree, Strongly agree
5	If it is possible to cure people with fatal diseases by gene therapy, how much do you agree that those people ought to be allowed to do this?	Strongly disagree, Disagree, Mildly disagree, Neither agree nor disagree, Mildly agree, Agree, Strongly agree
6	If it is possible to cure people with debilitating diseases*, such as Alzheimer's dementia, and Parkinson's disease, by gene therapy, how much do you agree that those people ought to be allowed to do this?<br>*Debilitating disease: those with debilitating disease can no longer perform daily functions like eating or bathing.	Strongly disagree, Disagree, Mildly disagree, Neither agree nor disagree, Mildly agree, Agree, Strongly agree
7	If you have a child with a usually fatal genetic disease, such as Down Syndrome, sickle cell anemia, muscular dystrophy, willing to have child undergo gene therapy to have their genes corrected?	Strongly disagree, Disagree, Mildly disagree, Neither agree nor disagree, Mildly agree, Agree, Strongly agree
8	If gene therapy is able to change a child's inherited characteristics by changing the child's genetic structure in the womb before they were born and you were making the decision, would consider doing so to improve his/her general physical health?	Strongly disagree, Disagree, Mildly disagree, Neither agree nor disagree, Mildly agree, Agree, Strongly agree
9	If gene therapy is able to change parents' genes in order to have a smarter or better-looking child and you were making the decision, would consider to do so?	Strongly disagree, Disagree, Mildly disagree, Neither agree nor disagree, Mildly agree, Agree, Strongly agree
10	Do you agree with that there is a reasonable chance that gene therapy will become a common treatment modality over the next few years?	Strongly disagree, Disagree, Mildly disagree, Neither agree nor disagree, Mildly agree, Agree, Strongly agree
11	Do you agree with that Chinese government ought to fund scientific research on developing new gene therapy treatment?	Strongly disagree, Disagree, Mildly disagree, Neither agree nor disagree, Mildly agree, Agree, Strongly agree

12	Do you agree with that Chinese government ought to approve gene therapy treatments for use in China?	Strongly disagree, Disagree, Mildly disagree, Neither agree nor disagree, Mildly agree, Agree, Strongly agree
13	What is your main concern in terms of gene therapy applied in humans? (multiple choices)	Passing genetic changes to offspring; High cost; Adverse medical side effects; Privacy; Going against nature; Going against religious belief
14	What is your sex?	Male or female
15	What is your age?	<18, 18-30, 30-40, 40-50, >50
16	Which municipality/province/autonomous regions of China do you live now?	Ask participants to write down
17	How long do you live there?	<3 year, 3-5 years, >5 years
18	Do you have a religious believe?	Yes/No
19	What is your highest level of education qualification?	Primary school or below, Middle school, High school, Undergraduate, Postgraduate or above
20	Do you have children?	Yes/No
21	Employment Status: Are you currently…?	Employed for wages, Self-employed, Out of work and looking for work, Out of work but not currently looking for work, A homemaker, A student, Military, Retired, Unable to work
21.1	Have you ever worked at a medical or health related field?	Yes/No
21.2	What is your profession at a medical or health related field?	Medical doctor, Scientific researcher, Nurse, Allied health worker, Other role at hospital/medical centre, Other (Please specify)
22	How long have you even worked at a medical or health related field?	< 3 years, 3-5 years, >5 years
23	Compare with the average level of wealth in the city/area you are living in, how would	Far below average wealth, Below average

	you describe your family financial situation?	wealth, Average wealth, Above average wealth, Far above average wealth
24	Do you or anyone you know have one of conditions below?	Inherited diseases, such as Down syndrome, sickle cell anemia, or muscular dystrophy;<br>Debilitating disease, such as Alzheimer's dementia, or Parkinson's disease;<br>No

Table S2. Respondent demographics of clinician (N = 2165) and the public (N = 11036) groups.

Characteristic	Clinicians	The public	χ^2 (*p* value)
**Gender, no. (%)**			3.602 (0.058)
Male	957 (44.2)	4635 (42.0)	
Female	1208 (55.8)	6401 (58.0)	
**Age, no. (%)**			691.157 (0.000)
Below 18	5 (0.2)	221 (2.0)	
18-25	270 (12.5)	4148 (37.6)	
26-30	790 (36.5)	2423 (22.0)	
31-40	797 (36.8)	2464 (22.3)	
41-50	224 (10.3)	1278 (11.6)	
51-60	69 (3.2)	449 (4.1)	
Above 60	10 (0.5)	53 (0.5)	
**Educational level, no. (%)**			2283.017 (0.000)
Primary school or below	11 (0.5)	48 (0.4)	
Middle school	7 (0.3)	480 (4.3)	
High school	27 (1.2)	1193 (10.8)	
Bachelor	924 (42.7)	8000 (72.5)	
Postgraduate or above	1196 (55.2)	1315 (11.9)	
**Residence*, no. (%)**			13.342 (0.004)
Mainland China	2077 (95.9)	10731 (97.2)	
Hong Kong and Macau	88 (4.1)	305 (2.8)	
**Religion, no. (%)**			0.345 (0.557)
Religious	361 (16.7)	1784 (16.2)	
Not religious	1804 (83.3)	9252 (83.8)	
**Having children, no. (%)**			61.044 (0.000)
Yes	1158 (53.5)	4893 (44.3)	

No	1007 (46.5)	6143 (55.7)	
**Having friends/relatives afflicted with a genetic disease, no. (%)**			299.317 (0.000)
Yes	1652 (76.3)	6219 (56.4)	
No	513 (23.7)	4817 (43.6)	

*Residence was re-organized as mainland China, Hong Kong and Macau. Data did not include Taiwan.

Note: We also examined whether the two groups had significant differences in such demographic variables by employing Chi-square tests.

Table S3. Summary of scores of respondents rated each question in clinician and the public groups.

**Questions (Q)**	**Clinicians, mean (*SD*)**	**The public, mean (*SD*)**	***t* (*p* value)**
Q1. (Five-likert points)	4.350 (0.749)	4.168 (0.835)	9.421 (0.000)
Q2. (Five-likert points)	3.671 (0.980)	2.783 (1.147)	37.419 (0.000)
Q3. (Seven-likert points)	5.081 (1.335)	4.815 (1.279)	8.770 (0.000)
Q4. (Seven-likert points)	4.368 (1.479)	4.260 (1.376)	3.146 (0.002)
Q5. (Seven-likert points)	5.802 (1.252)	5.592 (1.338)	7.037 (0.000)
Q6. (Seven-likert points)	5.717 (1.344)	5.467 (1.430)	7.840 (0.000)
Q7. (Seven-likert points)	5.998 (1.215)	5.736 (1.315)	9.028 (0.000)
Q8. (Seven-likert points)	4.726 (1.813)	4.704 (1.723)	0.517 (0.605)
Q9. (Seven-likert points)	3.770 (1.849)	4.092 (1.812)	-7.429 (0.000)
Q10. (Seven-likert points)	4.277 (1.553)	4.466 (1.417)	-5.260 (0.000)
Q11. (Seven-likert points)	5.187 (1.339)	5.022 (1.358)	5.226 (0.000)
Q12. (Seven-likert points)	4.840 (1.332)	4.655 (1.333)	5.915 (0.000)
Q13. Number shown in %			$\chi 2$ (p value)
passing genetic changes to offspring	1344 (62.1%)	6101 (55.3%)	33.992 (0.000)
high cost	1304 (60.2%)	6834 (61.9%)	2.196 (0.138)
adverse medical side effects	1491 (68.9%)	7941 (72.0%)	8.455 (0.004)
privacy	571 (26.4%)	3130 (28.4%)	3.544 (0.060)
going against nature	1536 (70.9%)	6621 (60.0%)	91.957 (0.000)
going against religious belief	285 (13.2%)	869 (7.9%)	63.483 (0.000)

Note: We conducted a series of independent samples t test to explore the differences in the answers between two groups.

Table S4. Summary of respondents' attitude to using gene therapy in children that affected by if having children. In order to examine whether having children or not has influences on participants' attitudes and cognitions to gene therapy, independent samples t tests were also employed. Results were presented in the following table.

Questions	**Clinicians (*N* = 2165)**		
	Without children, mean (*SD*)	With children, mean (*SD*)	*t* (*p* value)
Q7	5.912 (1.196)	6.073 (1.226)	-3.081 (0.002)
Q8	4.681 (1.740)	4.765 (1.874)	-1.079 (0.281)
Q9	3.742 (1.797)	3.794 (1.893)	-0.663 (0.507)
	**The public (*N* = 11036)**		
Q7	5.700 (1.294)	5.783 (1.339)	-3.356 (0.001)
Q8	4.659 (1.675)	4.761 (1.780)	-3.051 (0.002)
Q9	3.94 (1.749)	4.28 (1.872)	-9.725 (0.000)

Note: Children: 0 = Yes, 1 = No.

Table S5. Summary of standardized beta coefficients (β) from multiple linear regression analyses used for determining association of demographics and the respondents' attitudes towards gene therapy. By treating participants' demographic information (i.e., gender, age, educational levels, religion, income levels, and having friends or relatives afflicted with a genetic disease) as independent variables, and scores on each question as dependent variables, several multiple linear regression analyses were conducted in two sub-samples. Results revealed the associations between demographic variations and participants' attitudes towards the gene therapy. In each model, gender, religion and having friends/relatives afflicted with a genetic disease are dummy variables, while age, educational levels and income levels are considered to be continuous. F tests tell the significance of the whole model. When the result of F test is significant, we can move to the results of individual tests (i.e., standardized beta coefficients and the corresponding p values). The importance as well as the influential direction of each predictor can be known from the absolute values and positivity/negativity of standardized beta coefficients, respectively.

	**Clinicians (*N* = 2165)**																							
	Q1		Q2		Q3		Q4		Q5		Q6		Q7		Q8		Q9		Q10		Q11		Q12	
	β	*p*	β	*p*	β	*p*	β	*p*	β	*p*	β	*p*	β	*p*	β	*p*	β	*p*	β	*p*	β	*p*	β	*p*
Gender	.030	.160	-.030	.140	.095	.000	.015	.480	.020	.352	.010	.627	.058	.006	.086	.000	-.002	.944	.083	.000	.028	.194	-.002	.935
Age	.049	.027	.020	.363	.058	.010	.018	.428	.034	.132	.043	.057	.018	.419	-.013	.557	.007	.758	.071	.002	.068	.003	.078	.001
Education	.164	.000	.290	.000	.107	.000	.050	.021	.123	.000	.155	.000	.171	.000	.032	.144	-.010	.652	.016	.463	.091	.000	.048	.028
Religion	-.026	.210	-.018	.392	-.028	.193	.019	.372	-.041	.055	-.029	.169	-.045	.034	-.004	.870	.006	.785	.013	.547	-.059	.005	-.035	.103
Income	.096	.000	.058	.007	.040	.075	-.018	.430	.043	.054	.082	.000	.067	.002	.069	.002	.018	.427	.038	.096	.049	.030	.039	.084
Diseased	.091	.000	.060	.004	.065	.002	-.030	.163	.062	.004	.046	.034	.075	.000	.029	.185	.005	.803	.026	.226	.090	.000	.049	.024
	F	sig	*F*	sig	*F*	sig	*F*	sig	*F*	sig	*F*	sig	*F*	sig	*F*	sig	*F*	sig	*F*	sig	*F*	sig	*F*	sig
	21.095	.000	38.607	.000	11.999	.000	1.441	.195	9.824	.000	15.054	.000	18.566	.000	5.092	.000	.232	.966	5.690	.000	11.928	.000	6.171	.000
	**The public (*N* = 11036)**																							
Gender	-.017	.060	-.087	.000	.047	.000	.020	.038	.005	.586	.008	.400	.021	.022	.070	.000	.038	.000	.042	.000	.015	.108	.012	.214
Age	.096	.000	-.094	.000	-.016	.120	.002	.850	.031	.002	.071	.000	.020	.049	.012	.228	.075	.000	.074	.000	.069	.000	.098	.000
Education	.172	.000	.125	.000	.033	.001	.027	.006	.049	.000	.053	.000	.097	.000	.007	.501	-.091	.000	-.069	.000	.011	.241	-.012	.217
Religion	.005	.573	.005	.620	-.047	.000	.017	.080	-.033	.000	-.034	.000	-.043	.000	.009	.332	.017	.065	-.002	.859	-.031	.001	-.003	.713
Income	.049	.000	.028	.003	.036	.000	.010	.329	.032	.001	.042	.000	.019	.052	.029	.003	.013	.165	.017	.087	.035	.000	.020	.039

Diseased	.114	.000	.041	.000	.071	.000	-.003	.761	.104	.000	.110	.000	.124	.000	.048	.000	-.015	.114	.020	.042	.087	.000	.052	.000
	F	sig	*F*	sig	*F*	sig	*F*	sig	*F*	sig	*F*	sig	*F*	sig	*F*	sig	*F*	sig	*F*	sig	*F*	sig	*F*	sig
	120.039	.000	69.497	.000	23.304	.000	2.731	.012	35.389	.000	52.272	.000	58.397	.000	16.940	.000	33.567	.000	27.506	.000	34.109	.000	29.947	.000

Note: Standardized beta coefficients are reported. Income levels: Mean = 3.00, SD = 0.742 (clinician); Mean = 2.89, SD = 0.740 (the public).
Gender: 0 = Male, 1 = Female; Religion: 0 = Religious, 1 = Not religious; Having friends or relatives afflicted with a genetic disease: 0 = Yes, 1 = No.

SUPPLEMENTAL FIGURES

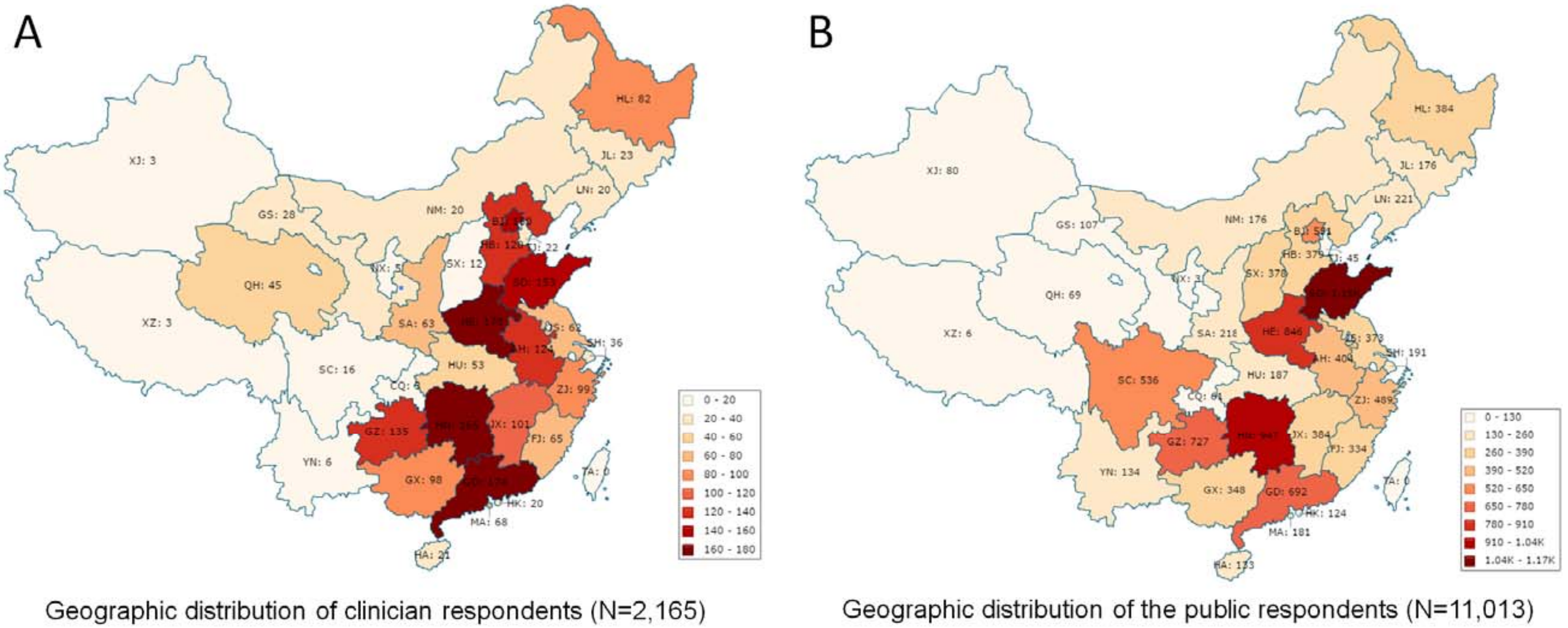

Figure S1. Geographic distributions of valid respondents across China. (A) Geographic distribution of clinician respondents (N=2,165) (B) Geographic distribution of the public respondents (N=11,013). Respondents from both groups were proportionally distributed across China based on the population distribution in each province.

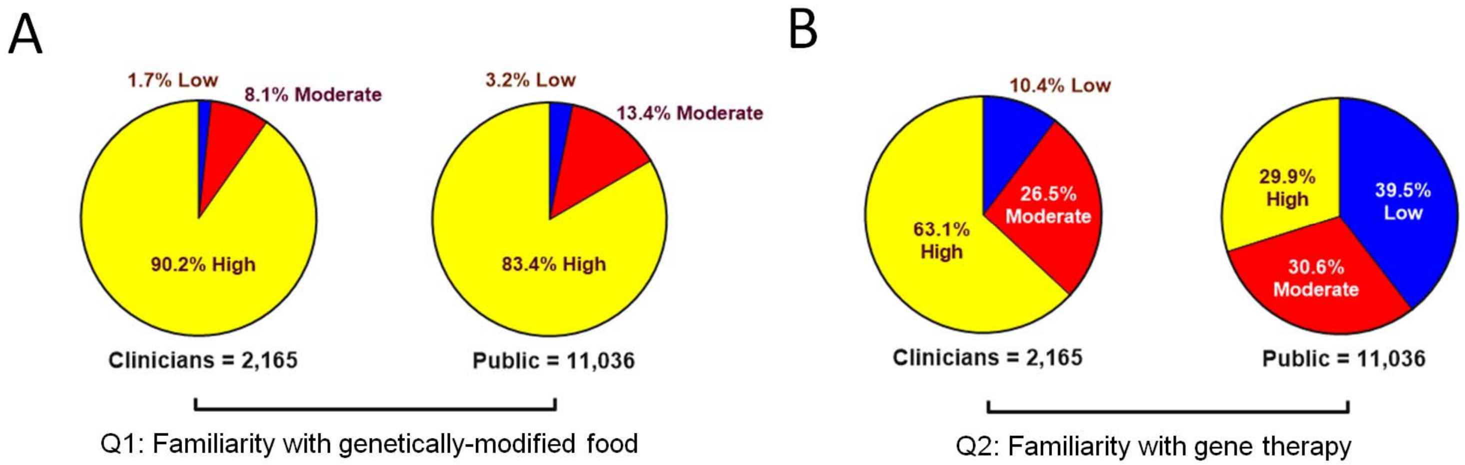

Figure S2. Proportion of respondents being familiar with genetically-modified food and gene therapy. (A) Proportion of respondents being familiar with genetically-modified food. Both the clinicians and public respondents showed the significantly high familiarity with GM food (90.2 % and 83.4%, respectively). (B) Proportion of respondents being familiar with gene therapy. The majority of clinicians (63.1%) had the remarkably higher familiarity with gene therapy than the public (29.9%). High represents the respondents were highly familiar with the term. Low represents the respondents were less familiar with the term. Moderate familiarity is in the between.

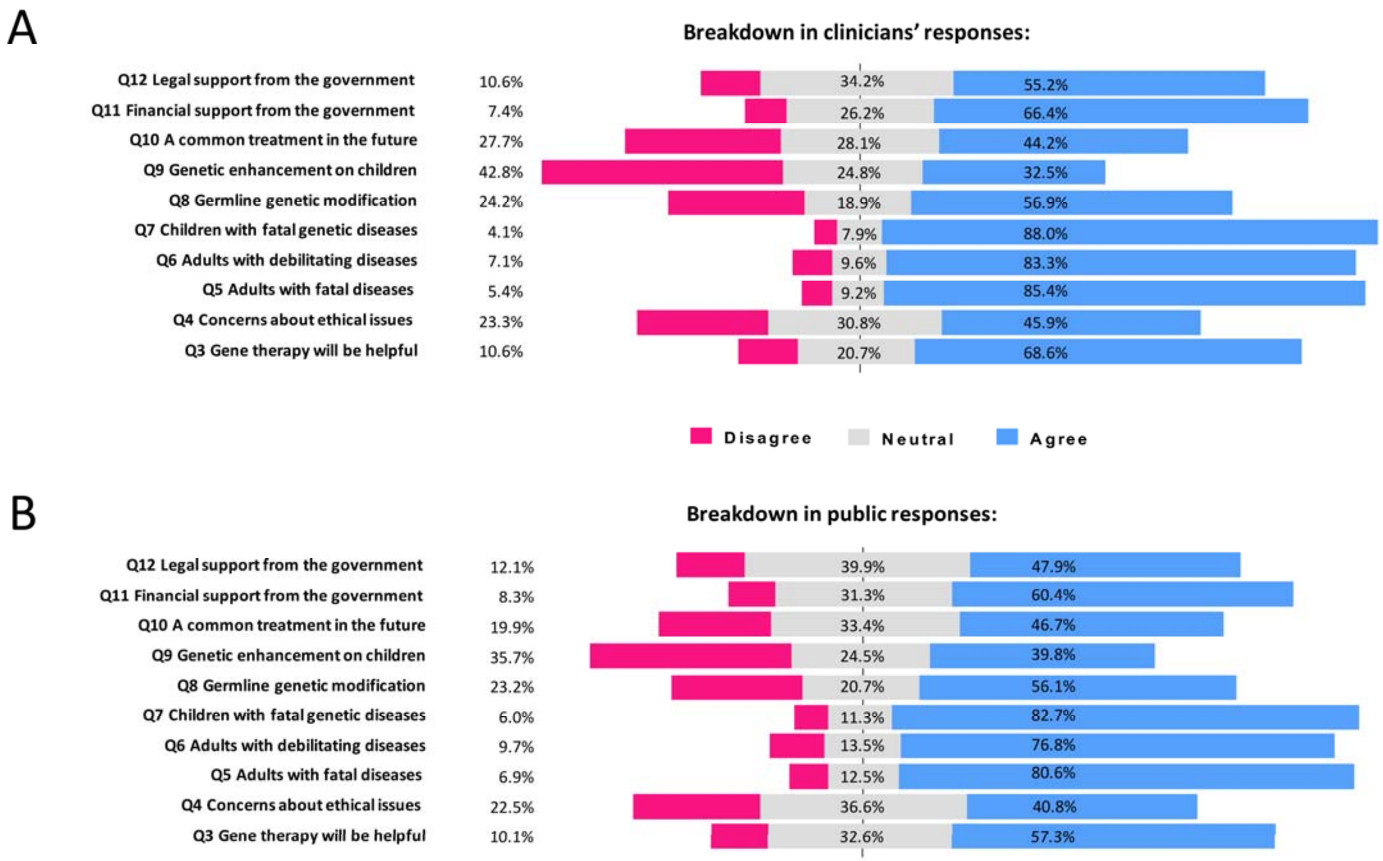

Figure S3. The proportion of clinicians' and public attitudes toward gene therapy. Responders who selected strongly disagree, disagree, mildly disagree for the individual question were merged to the population with the attitude of a disagreement. Responders who selected mildly agree, agree, strongly agree for the individual question were merged to the population with the attitude of an agreement.

SUPPLEMENTAL REFERENCES

1. Wang, YX, and Li, YT (2016). AME survey-003 A2: on the attractiveness of an medicine career in current China with a survey of 7,508 medical professionals and 443 non-medical professionals. *Quantitative imaging in medicine and surgery* **6**: 84-102.
2. McCaughey, T, Sanfilippo, PG, Gooden, GE, Budden, DM, Fan, L, Fenwick, E, *et al.* (2016). A Global Social Media Survey of Attitudes to Human Genome Editing. *Cell stem cell* **18**: 569-572.
3. Blendon, RJ, Gorski, MT, and Benson, JM (2016). The Public and the Gene-Editing Revolution. *The New England journal of medicine* **374**: 1406-1411.
4. Xiang, L, Xiao, L, Gou, Z, Li, M, Zhang, W, Wang, H, *et al.* (2015). Survey of Attitudes and Ethical Concerns Related to Gene Therapy Among Medical Students and Postgraduates in China. *Human gene therapy* **26**: 841-849.
