# Supplementary material for: Public Attitudes toward Gene Therapy in China
Source: Mol Ther Methods Clin Dev. 2017 Jun 20;6:40–2. doi: 10.1016/j.omtm.2017.05.008 (PMC5480269; doi:10.1016/j.omtm.2017.05.008)
Supplement: Document S2. Article plus Supplemental Information [file mmc2.pdf]

Molecular Therapy
Methods & Clinical Development
Commentary

# Public Attitudes toward Gene Therapy in China

Jiang-Hui Wang,[1,2,21] Rong Wang,[3,21] Jia Hui Lee,[1] Tiara W.U. Iao,[4] Xiao Hu,[5] Yu-Meng Wang,[4] Lei-Lei Tu,[6] Yi Mou,[7] Wen-Li Zhu,[8] Ai-Yong He,[9] Shen-Yu Zhu,[10] Di Cao,[4] Lei Yang,[11] Xiao-Bo Tan,[12] Qing Zhang,[8] Guan-Lu Liang,[13] Shu-Min Tang,[4] Ye-Di Zhou,[14] Li-Jun Feng,[15] Li-Jun Zhan,[16] Nan-Nan Tian,[8] Ming-Jie Tang,[17] Ya-Ping Yang,[18] Moeen Riaz,[1,2] Peter van Wijngaarden,[1,2] Gregory J. Dusting,[1,2] Guei-Sheung Liu,[1,2,19,20] and Yan He[14,20]

http://dx.doi.org/10.1016/j.omtm.2017.05.008

Gene therapy, a medical procedure that delivers genetic materials into a person to potentially prevent or treat disease at its genetic roots, has long fascinated scientists, clinicians, and the general public. Although gene therapy has a checkered history, with several prominent adverse events in early trials, significant safety and efficacy improvements in the last two decades have catapulted the technology back to the center stage of medical research. As gene therapy rapidly progresses toward widespread clinical use, there is growing evidence of concern and skepticism in the scientific community and the general public alike.[1] Among a range of concerns about gene therapy, balancing the benefits and risks of gene therapy stands out as a recurring theme. Ethical debate over the use of gene therapy for non-medical purposes, such as genetic enhancement of intelligence or physical aesthetics, has been robust. In addition, recurrent concerns have been raised about the ethical implications of gene therapy on human germlines.

Numerous studies of public attitudes to gene therapy or gene editing have been carried out in the past; however, these studies differ widely in their methods and the demographic and geographic attributes of the populations surveyed.[1,2] Of note, most of these studies have been conducted in Western countries. Given that China is the most populous nation in the world and has rapidly expanding capacity in gene therapy research, it is of utmost importance to understand the attitudes of the Chinese public and clinicians in relation to the application of gene therapy.

Here we performed an online survey from 13,563 participants across China to explore attitudes toward gene therapy in different contexts (the complete questionnaire can be seen in Table S1). The majority of respondents completed all of the questions in the survey, giving a response rate of 97.3% (n = 13,201/13,563). Of valid respondents, 16.4% (n = 2,165/13,201) and 83.6% (n = 11,036/13,201) were clinicians (ascertained by self-report) and members of the general public, respectively. More than half of the respondents from both the clinician and general public groups were female (55.8% and 58.0%, respectively), and respondents' ages ranged from 18 to 50 years. Other demographic information is provided in Table S2. We analyzed the geographic distributions of respondents of the two participant pools (the clinicians and the members of general public; Figure S1) and found that respondents from both groups were proportionally distributed across China based on the population distribution in each province.[3]

Several important findings have been obtained from our study. First of all, although gene therapy has been a familiar theme in the medical research community for decades, our study showed that both clinicians and members of the general public have much less awareness of gene therapy (63.1% and 29.9%, respectively) than genetically-modified (GM) food (90.2% and 83.4%, respectively; Q1 and Q2, Table S3 and Figure S2), a gene technology of which the public generally has a greater awareness and usually has been selected to compare with new technology previously.[2] These findings are consistent with the results of other studies: Blendon et al.[1] found that only 31% of the general public in the United States was familiar with gene therapy. The gap in awareness of gene therapy and GM food is likely related to the comparatively limited coverage of gene therapy in the media over the last few decades. In contrast to gene therapy, GM food is also perhaps more tangible

[1]Centre for Eye Research Australia, Royal Victorian Eye and Ear Hospital, East Melbourne, VIC 3002, Australia; [2]Ophthalmology, Department of Surgery, University of Melbourne, East Melbourne, VIC 3002, Australia; [3]College of Management, Shenzhen University, Shenzhen, Guangdong 518060, China; [4]Department of Ophthalmology and Visual Sciences, The Chinese University of Hong Kong, Hong Kong; [5]School of Marxism, Central South University, Changsha, Hunan 410011, China; [6]Department of Ophthalmology, The First Affiliated Hospital of Jinan University, Guangzhou, Guangdong 510630, China; [7]Department of Ultrasound, Guizhou Provincial People's Hospital, Guiyang, Guizhou 550002, China; [8]Eye Hospital of China Academy of Chinese Medical Sciences, Beijing 100040, China; [9]Department of Orthopaedics, The Second Xiangya Hospital, Central South University, Changsha, Hunan 410011, China; [10]Department of Cardiothoracic Surgery, First Affiliated Hospital of Gannan Medical University, Ganzhou, Jiangxi 341000, China; [11]Department of Internal Medicine, Gaochun People's Hospital, Nanjing, Jiangsu 211300, China; [12]Department of Ophthalmology, Affiliated Hospital of Chengde Medical University, Chengde, Hebei 067000, China; [13]Department of Ophthalmology, Zhejiang Hospital, Hangzhou, Zhejiang 310000, China; [14]Department of Ophthalmology, The Second Xiangya Hospital, Central South University, Changsha, Hunan 410011, China; [15]Department of Otorhinolaryngology, Head and Neck Surgery, Affiliated Xingtan Hospital of The First People's Hospital of Shunde, Foshan, Guangdong 528313, China; [16]Department of Urology, The Central Hospital of Enshi Autonomous Prefecture, Enshi, Hubei 445000, China; [17]Department of General Surgery, Qinghai Provincial People's Hospital, Xining, Qinghai 810000, China; [18]Department of Ophthalmology, Eye and ENT Hospital of Fudan University, Shanghai 200031, China; [19]Menzies Institute for Medical Research, University of Tasmania, Hobart, Tasmania 7000, Australia
[20]These authors contributed equally to this work.
[21]These authors contributed equally to this work.

**Correspondence:** Guei-Sheung Liu, Menzies Institute for Medical Research, University of Tasmania, Hobart, Tasmania 7000, Australia.
**E-mail:** rickliu0817@gmail.com
**Correspondence:** Yan He, Department of Ophthalmology, The Second Xiangya Hospital, Central South University, Changsha, Hunan 410011, China.
**E-mail:** yanhe6416@csu.edu.cn

 © 2017 The Author(s).
This is an open access article under the CC BY license (http://creativecommons.org/licenses/by/4.0/).

to both clinicians and the general public because it is commonly encountered in supermarkets and the farming and containment of GM crops has gained media attention. These results may reflect that, while gene therapy is a prominent theme in the research community, it has not yet gained attraction with clinicians and medical students.

Here we also show that both clinicians and the general public mildly agreed that gene therapy would be beneficial for improving human health in the future, and clinicians responded more optimistically to this question than respondents from the general public (Q3, Table S3 and Figure S3). Interestingly, although both groups agreed on the promise of gene therapy as a future medical treatment, clinicians appeared to be more conservative than the public when asked if gene therapy would be a common therapy over the next few years (Q10, Table S3 and Figure S3). Moreover, both clinicians and the public strongly support the use of gene therapy to treat fatal or debilitating diseases in adults and fatal disease in children (Q5–Q8; Table S3 and Figure S3). Interestingly, we observed that respondents were more supportive of the use of gene therapy for fatal diseases than debilitating diseases, such as Alzheimer's dementia and Parkinson's disease. This is a trend that has also been described in other studies,[4] and it may be attributed to the perception that gene therapy is not without significant risks and that, at this stage, the risk-benefit ratio of using gene therapy for medical conditions is perceived as inversely proportional to the severity of the disease.

In addition, there was substantially less support from both clinicians and members of the general public for the use of gene therapy for genetic enhancement for non-medical purposes, such as increasing intelligence and physical attributes (Q9, Table S3 and Figure S3). Results from previous studies support this finding.[1,2] Of note, we found that members of the general public were only neutral toward genetic enhancement, while clinicians disagreed with this application of gene therapy. Clinicians were less supportive of non-medical genetic enhancement, regardless of whether or not they had children. In contrast, members of the general public with children were more amenable to the idea than were those without (Table S4). The relative acceptance of genetic enhancement among members of the general public in China may reflect broader sociocultural pressures to excel in an increasingly competitive world.[5] This finding draws attention to the need for political debate and legislative action to regulate the scope of non-medical genetic enhancement in China.

Indeed, these medical and ethical issues are the main concerns raised by clinicians and public respondents in our study (Q13, Table S3 and Figure S3). Our results showed that clinicians are more concerned about gene therapy going against nature (70.9%), followed by adverse medical side effects (68.9%), whereas the public respondents were primarily concerned about adverse medical side effects of gene therapy (72.0%), followed by high cost (61.9%). Interestingly, while both respondent groups were concerned about the safety of gene therapy, only clinicians were more concerned about gene therapy going against nature. This may mean that the public may not have as great an understanding as clinicians about the full implications and potential of gene therapy. Moreover, this may reflect the fact that the broader implications of gene therapy may not have been widely discussed in public due to a culture that does not always encourage freedom of debate on controversial subjects. Accordingly much of the debate about the ethics of gene therapy and editing in humans over the last three decades has come from commentators in Western countries.[1,6] Despite the concerns of clinicians about the "unnatural" nature of gene therapy, our results showed that both clinicians and the public were nearly neutral when asked if gene therapy will raise ethical issues (Q4, Table S3 and Figure S3). Indeed, the relatively loose regulation of the ethical review of clinical trials in China's medical community means that gene therapy has been able to be more extensively researched in China compared to other countries. Although this is likely to have, in part, accelerated China's position as a leading country in gene therapy research in humans compared to other countries with strict ethical regulations,[7] we reflect that it is important to ensure that both clinicians and the general public in China are well-informed of the implications of gene therapy and that careful ethical consideration is given to this research to uphold both safety and core human rights.

Our study showed that attitudes and perceptions of gene therapy were influenced by specific demographic factors (see Tables S4 and S5). We found that women were significantly more likely than men to accept gene therapy for use in children with inherited diseases and in germline cells. This differs from the results of a study that showed men were found to be more accepting of all applications of gene editing compared to females.[2] We also found that clinicians and public respondents with higher education or higher self-reported income were more likely to be supportive of the use of gene therapy for severe diseases. This result is consistent with a report that suggests people from developed countries with higher gross domestic product (GDP) per capita were more supportive of all health-related applications of gene editing.[2] Respondents with self-reported religious affiliations were more likely to be significantly against using gene therapy to treat genetic diseases and were notably more reluctant to support government funding for gene therapy research. These results are perhaps not surprising, given that many religions have conservative positions in relation to other scientific developments, including human embryonic stem cell research. As expected, respondents with personal knowledge of an individual with a fatal debilitating or inherited disease were more accepting of the use of gene therapy for these conditions.

This study regarding the public perception of gene therapy was conducted via an online survey through social media, which can be used as a powerful tool to engage the public in biomedical research. However, there are several limitations to this study as a result of this method. First, there are many who do not have access to the internet or social media in China, and thus it is inevitable that our study has perhaps missed specific

population groups, in particular, older individuals or those from regional or rural areas.[8] Second, the use of the phrase "gene therapy" in the questions deals with non-therapeutic applications, such as genetic enhancement, which might potentially lead to an undesirable response because the "therapeutic tone" usually represents a positiveness. Third, a few participants in the general public group might have medical backgrounds that could have influenced their responses to the questions. Fourth, although we sampled respondents from different provinces across China in this survey, it may not absolutely reflect the Chinese population as a whole. For example, 84.4% of the public respondents in our study had at least a bachelor's degree, which is a far higher proportion than in the general public,[9] suggesting that there may be some recruitment bias. Of note, there were a few participants in the clinician group who did not have a college degree, suggesting that they were likely "barefoot doctors" who received basic medical training at county level to provide primary care to village populations.[10] Follow-up studies using qualitative methods, such as focus groups and interviews, will be necessary to explore the attitudes of other stakeholders in greater depth.

In summary, our study is the first to investigate the attitudes of clinicians and members of the general public toward gene therapy in China. Our findings highlight the lack of knowledge of gene therapy among a large proportion of the public as well as around one-third of clinicians in China. Both groups were wary about using gene therapy for germline cells. However, the public was more amenable to genetic enhancement for non-medical reasons than clinicians. The safety of gene therapy was among the primary concerns for both the clinicians and public of China. Our results indicate that there is a need for both clinicians and the public to be more aware of the progress of gene therapy and its implications in order to keep the potential providers and receivers of this therapy well-informed. It also highlights the need for more ethical discussion regarding the uses of gene therapy from both China's medical community and the general public to guide law and policy-making and the safe translation of gene therapy to the clinical setting.

## SUPPLEMENTAL INFORMATION

Supplemental Information includes Supplemental Materials and Methods, three figures, and five tables and can be found with this article online at http://dx.doi.org/10.1016/j.omtm.2017.05.008.

## CONFLICTS OF INTEREST

The authors declare no conflicts of interest.

## ACKNOWLEDGMENTS

This work was supported by the National Natural Science Foundation of China (#81600714). J.-H.W. is the recipient of a R.B. McComas Research Scholarship in Ophthalmology, a Gordon P. Castles Scholarship, and a Melbourne Research Scholarship. P.v.W. is the recipient of a University of Melbourne, Annemarie Mankiewicz-Zelkin Fellowship and a Clinical Investigator Award from the Sylvia & Charles Viertel Charitable Foundation. G.J.D. is the recipient of a National Health & Medical Research Council Research Fellowship (#1003113). The Centre for Eye Research Australia receives operational infrastructure support from the Victorian Government. We are grateful for the assistance of Alex W. Hewitt, Yi-Xiang Wang, Lei Xu, Yuan Zhao, Cheng-Liang Zheng, Yan-Jiang Liu, Cong Ye, Cheng Xie, Cheng-Yan Chen, Hong Chen, Tong Wu, Li-Ming Dai, Yuan Jiang, Hui Li, Xia Lan, Xu Liang, Ting-Ting Mo, Jie Ren, Shu-Min Bao, Ling Shen, Kun-Xiu Song, Xiang-Qian Wang, Xin-Wen Wang, Xiang-Tao Xie, Yu-Hua Luo, Rong-Mou Zhang, Kuan He, Fei Hu, and Bo-Xiang Zhang.

## REFERENCES

1. Blendon, R.J., Gorski, M.T., and Benson, J.M. (2016). The Public and the Gene-Editing Revolution. N. Engl. J. Med. *374*, 1406–1411.
2. McCaughey, T., Sanfilippo, P.G., Gooden, G.E., Budden, D.M., Fan, L., Fenwick, E., Rees, G., MacGregor, C., Si, L., Chen, C., et al. (2016). A Global Social Media Survey of Attitudes to Human Genome Editing. Cell Stem Cell *18*, 569–572.
3. Wang, L., and Chen, L. (2016). Addendum: Spatiotemporal dataset on Chinese population distribution and its driving factors from 1949 to 2013. Sci. Data *3*, 160078.
4. Condit, C.M. (2010). Public attitudes and beliefs about genetics. Annu. Rev. Genomics Hum. Genet. *11*, 339–359.
5. Li, M., Xue, H., Wang, W., and Wang, Y. (2017). Parental Expectations and Child Screen and Academic Sedentary Behaviors in China. Am. J. Prev. Med. *52*, 680–689.
6. Mariscal, C., and Petropanagos, A. (2016). CRISPR as a driving force: the Model T of biotechnology. Monash Bioeth. Rev. *34*, 101–116.
7. Cyranoski, D. (2016). CRISPR gene-editing tested in a person for the first time. Nature *539*, 479.
8. Fong, M.W. (2009). Digital divide between urban and rural regions in China. Electron J Inf Syst Dev Ctries. *36*, 1–12.
9. Yu, K., Stith, A.L., Liu, L., and Chen, H. (2012). Global Perspectives on Higher Education: Tertiary education at a glance China, *Volume 24* (Sense Publishers, Rotterdam).
10. Wong, W.C., Jiang, S., Ong, J., Peng, M., Wan, E., Zhu, Z., Lam, C.L., Kidd, M., and Roland, M. (2016). Availability and use of primary care facilities in China: a nationwide representative survey. Lancet *388* (*Suppl 1*), S18.

**OMTM, Volume *6***

## Supplemental Information

# Public Attitudes toward Gene Therapy in China

**Jiang-Hui Wang, Rong Wang, Jia Hui Lee, Tiara W.U. Iao, Xiao Hu, Yu-Meng Wang, Lei-Lei Tu, Yi Mou, Wen-Li Zhu, Ai-Yong He, Shen-Yu Zhu, Di Cao, Lei Yang, Xiao-Bo Tan, Qing Zhang, Guan-Lu Liang, Shu-Min Tang, Ye-Di Zhou, Li-Jun Feng, Li-Jun Zhan, Nan-Nan Tian, Ming-Jie Tang, Ya-Ping Yang, Moeen Riaz, Peter van Wijngaarden, Gregory J. Dusting, Guei-Sheung Liu, and Yan He**

**SUPPLEMENTAL MATERIALS**

**Ethical Review**

This study was reviewed and approved by Human Subjects Research Ethics Board of the Second Xiangya Hospital, Central South University, China. All respondents gave informed consent prior to the survey and agreed to the use of their deidentified data for research purposes.

**Study participants and survey dissemination**

Clinicians and members of general public across China were invited to participate in a cross-sectional survey about attitudes to gene therapy. The survey was placed on Sojump platform (www.sojump.com) during the period from August 24 to November 2, 2016 to attract responses from members of the general population. The survey was promoted via the social media platform Wechat (https://wx.qq.com/) as well as other online tools such as Weibo China's Twitter equivalent through personal contacts, friends, and colleagues with the aim to balance out the young age bias given the social-media-based recruitment[1, 2].The participation of clinicians was achieved through survey dissemination in a variety of hospitals and medical conferences by trained research coordinators across the country and via Wechat. A brief introduction described gene therapy as well as the objectives of the survey and participants were offered an opportunity to opt out of the survey. The survey was closed at 3pm November 2, 2016 (GMT+8).

**Survey instrument**

In the development of our survey, we reviewed the literature, formulated questions, and conducted a cognitive phase testing in ten participants to refine the questionnaire [2-4]. Subsequently, changes were made to ensure questionnaire was understandable by both participant groups. The survey

was designed to gauge knowledge of genetically-modified (GM) food and gene therapy by using a five-point Likert scale (rating from 1 = never heard of it to 5 = know it very well) or agreement for questions in relation to gene therapy by using a seven-point Likert scale (rating from 1 = strongly disagree to 7 = strongly agree). The final structured survey (Table S1) was divided into eight sections: 1) a brief introduction to gene therapy, serving as background information; 2) whether the participant has heard of genetically modified food or gene therapy (Question 1 and 2); 3) general attitude to gene therapy and relevant ethical issues (Question 3, 4, and 10); 4) attitude to gene therapy if used to treat adults with fatal or debilitating diseases (Question 5 and 6); 5) attitude to gene therapy if used to treat children with fatal genetic diseases or to germline genetic modification or to enhance their genetic properties (Question 7, 8, and 9); 6) attitude to funding and legal support from the government for development of gene therapy in China (Question 11and 12); 7) main concerns about gene therapy applied in humans (Question 13); 8) demographics of participants including age, gender, residence, education, religion, children, occupation and financial condition (Question 14-24).

**Data management and analysis**

We analyzed the data by employing SPSS 20.0. Respondents rated all the questions on a five-point Likert scale or a seven-point Likert scale. To test the mean differences in participants' answers, we conducted t test or one-way ANOVA, while multiple linear regressions (Table S2) were conducted to examine the influences of demographic variables on the ratings. We considered the results to be statistically significant when $p$ values were of <0 .05.

**SUPPLEMENTAL TABLES**

**Table S1.** A complete questionnaire is comprised of introduction.

<table>
<tr><td colspan="3">A short introduction to gene therapy, provided for informational purposes<br>Gene therapy is a therapeutic strategy that corrects defects in the genetic material of a human being. The diseases can be prevented or treated by gene therapy via replace or modify a faulty gene of a patient’s cells instead of using drugs or surgery. Gene therapy is currently only being used in clinic for the treatment of diseases that have no other cures. Gene therapy aims to eliminate the defect on the molecular level of the DNA via several approaches, including:<br>1. Replacing or correcting a mutated gene that causes disease with a healthy copy of the gene.<br>2. Inactivating, or “knocking out,” a mutated gene that is functioning improperly.<br>3. Introducing a new gene into the body to help fight a disease.<br>Although gene therapy is a promising treatment option for a number of diseases (including some inherited and non-inherited disorders, a few types of cancer), the technique remains risky and is still under study to make sure that it will be safe and effective. Despite the rapid development and tremendous investment in health care and medical research in the last two decades, China is still behind the developed countries in terms of research and development of gene therapy.<br>The questionnaire below is to survey the attitude of the public and clinicians towards gene therapy in China. It will take you 10 minutes or so to finish it. Your participation in this project is entirely voluntary. If you do not wish to take part in, you are not obliged to. All comments and responses will be treated confidentially. The data will only be used for current and future analyses to address various research questions.</td></tr>
<tr><td colspan="2">Questions (Q)</td><td>Answer options</td></tr>
<tr><td>1</td><td>Have you ever heard about genetically-modified food in the last 5 years?</td><td>Never heard of it, Head of it, Know a litter bit about it, Know a fair amount about it, Know it very well</td></tr>
<tr><td>2</td><td>Have you ever heard about gene therapy in the last 5 years?</td><td>Never heard of it, Head of it, Know a litter bit about it, Know a fair amount about it, Know it very well</td></tr>
<tr><td>3</td><td>Do you agree with that gene therapy will be helpful in addressing health needs of patients over the next few years?</td><td>Strongly disagree, Disagree, Mildly disagree, Neither agree nor disagree, Mildly agree,</td></tr>
</table>

| | | |
|---|---|---|
| | | Agree, Strongly agree |
| 4 | Do you agree with that gene therapy poses significant ethical issues in terms of altering the human genome*?<br>*Human genome is the complete set of genetic material for human. | Strongly disagree, Disagree, Mildly disagree, Neither agree nor disagree, Mildly agree, Agree, Strongly agree |
| 5 | If it is possible to cure people with fatal diseases by gene therapy, how much do you agree that those people ought to be allowed to do this? | Strongly disagree, Disagree, Mildly disagree, Neither agree nor disagree, Mildly agree, Agree, Strongly agree |
| 6 | If it is possible to cure people with debilitating diseases*, such as Alzheimer's dementia, and Parkinson's disease, by gene therapy, how much do you agree that those people ought to be allowed to do this?<br>*Debilitating disease: those with debilitating disease can no longer perform daily functions like eating or bathing. | Strongly disagree, Disagree, Mildly disagree, Neither agree nor disagree, Mildly agree, Agree, Strongly agree |
| 7 | If you have a child with a usually fatal genetic disease, such as Down Syndrome, sickle cell anemia, muscular dystrophy, willing to have child undergo gene therapy to have their genes corrected? | Strongly disagree, Disagree, Mildly disagree, Neither agree nor disagree, Mildly agree, Agree, Strongly agree |
| 8 | If gene therapy is able to change a child's inherited characteristics by changing the child's genetic structure in the womb before they were born and you were making the decision, would consider doing so to improve his/her general physical health? | Strongly disagree, Disagree, Mildly disagree, Neither agree nor disagree, Mildly agree, Agree, Strongly agree |
| 9 | If gene therapy is able to change parents' genes in order to have a smarter or better-looking child and you were making the decision, would consider to do so? | Strongly disagree, Disagree, Mildly disagree, Neither agree nor disagree, Mildly agree, Agree, Strongly agree |
| 10 | Do you agree with that there is a reasonable chance that gene therapy will become a common treatment modality over the next few years? | Strongly disagree, Disagree, Mildly disagree, Neither agree nor disagree, Mildly agree, Agree, Strongly agree |
| 11 | Do you agree with that Chinese government ought to fund scientific research on developing new gene therapy treatment? | Strongly disagree, Disagree, Mildly disagree, Neither agree nor disagree, Mildly agree, Agree, Strongly agree |

| | | |
|---|---|---|
| 12 | Do you agree with that Chinese government ought to approve gene therapy treatments for use in China? | Strongly disagree, Disagree, Mildly disagree, Neither agree nor disagree, Mildly agree, Agree, Strongly agree |
| 13 | What is your main concern in terms of gene therapy applied in humans? (multiple choices) | Passing genetic changes to offspring; High cost; Adverse medical side effects; Privacy; Going against nature; Going against religious belief |
| 14 | What is your sex? | Male or female |
| 15 | What is your age? | <18, 18-30, 30-40, 40-50, >50 |
| 16 | Which municipality/province/autonomous regions of China do you live now? | Ask participants to write down |
| 17 | How long do you live there? | <3 year, 3-5 years, >5 years |
| 18 | Do you have a religious believe? | Yes/No |
| 19 | What is your highest level of education qualification? | Primary school or below, Middle school, High school, Undergraduate, Postgraduate or above |
| 20 | Do you have children? | Yes/No |
| 21 | Employment Status: Are you currently…? | Employed for wages, Self-employed, Out of work and looking for work, Out of work but not currently looking for work, A homemaker, A student, Military, Retired, Unable to work |
| 21.1 | Have you ever worked at a medical or health related field? | Yes/No |
| 21.2 | What is your profession at a medical or health related field? | Medical doctor, Scientific researcher, Nurse, Allied health worker, Other role at hospital/medical centre, Other (Please specify) |
| 22 | How long have you even worked at a medical or health related field? | < 3 years, 3-5 years, >5 years |
| 23 | Compare with the average level of wealth in the city/area you are living in, how would | Far below average wealth, Below average |

| | | |
|---|---|---|
| | you describe your family financial situation? | wealth, Average wealth, Above average wealth, Far above average wealth |
| 24 | Do you or anyone you know have one of conditions below? | Inherited diseases, such as Down syndrome, sickle cell anemia, or muscular dystrophy;<br>Debilitating disease, such as Alzheimer's dementia, or Parkinson's disease;<br>No |

**Table S2**. Respondent demographics of clinician (N = 2165) and the public (N = 11036) groups.

| Characteristic | Clinicians | The public | $\chi^2$ (*p* value) |
|---|---|---|---|
| **Gender, no. (%)** | | | 3.602 (0.058) |
| Male | 957 (44.2) | 4635 (42.0) | |
| Female | 1208 (55.8) | 6401 (58.0) | |
| **Age, no. (%)** | | | 691.157 (0.000) |
| Below 18 | 5 (0.2) | 221 (2.0) | |
| 18-25 | 270 (12.5) | 4148 (37.6) | |
| 26-30 | 790 (36.5) | 2423 (22.0) | |
| 31-40 | 797 (36.8) | 2464 (22.3) | |
| 41-50 | 224 (10.3) | 1278 (11.6) | |
| 51-60 | 69 (3.2) | 449 (4.1) | |
| Above 60 | 10 (0.5) | 53 (0.5) | |
| **Educational level, no. (%)** | | | 2283.017 (0.000) |
| Primary school or below | 11 (0.5) | 48 (0.4) | |
| Middle school | 7 (0.3) | 480 (4.3) | |
| High school | 27 (1.2) | 1193 (10.8) | |
| Bachelor | 924 (42.7) | 8000 (72.5) | |
| Postgraduate or above | 1196 (55.2) | 1315 (11.9) | |
| **Residence*, no. (%)** | | | 13.342 (0.004) |
| Mainland China | 2077 (95.9) | 10731 (97.2) | |
| Hong Kong and Macau | 88 (4.1) | 305 (2.8) | |
| **Religion, no. (%)** | | | 0.345 (0.557) |
| Religious | 361 (16.7) | 1784 (16.2) | |
| Not religious | 1804 (83.3) | 9252 (83.8) | |
| **Having children, no. (%)** | | | 61.044 (0.000) |
| Yes | 1158 (53.5) | 4893 (44.3) | |

| | | | |
|---|---|---|---|
| No | 1007 (46.5) | 6143 (55.7) | |
| **Having friends/relatives afflicted with a genetic disease, no. (%)** | | | 299.317 (0.000) |
| Yes | 1652 (76.3) | 6219 (56.4) | |
| No | 513 (23.7) | 4817 (43.6) | |

*Residence was re-organized as mainland China, Hong Kong and Macau. Data did not include Taiwan.

Note: We also examined whether the two groups had significant differences in such demographic variables by employing Chi-square tests.

**Table S3.** Summary of scores of respondents rated each question in clinician and the public groups.

| **Questions (Q)** | **Clinicians, mean (*SD*)** | **The public, mean (*SD*)** | ***t* (*p* value)** |
|---|---|---|---|
| Q1. (Five-likert points) | 4.350 (0.749) | 4.168 (0.835) | 9.421 (0.000) |
| Q2. (Five-likert points) | 3.671 (0.980) | 2.783 (1.147) | 37.419 (0.000) |
| Q3. (Seven-likert points) | 5.081 (1.335) | 4.815 (1.279) | 8.770 (0.000) |
| Q4. (Seven-likert points) | 4.368 (1.479) | 4.260 (1.376) | 3.146 (0.002) |
| Q5. (Seven-likert points) | 5.802 (1.252) | 5.592 (1.338) | 7.037 (0.000) |
| Q6. (Seven-likert points) | 5.717 (1.344) | 5.467 (1.430) | 7.840 (0.000) |
| Q7. (Seven-likert points) | 5.998 (1.215) | 5.736 (1.315) | 9.028 (0.000) |
| Q8. (Seven-likert points) | 4.726 (1.813) | 4.704 (1.723) | 0.517 (0.605) |
| Q9. (Seven-likert points) | 3.770 (1.849) | 4.092 (1.812) | -7.429 (0.000) |
| Q10. (Seven-likert points) | 4.277 (1.553) | 4.466 (1.417) | -5.260 (0.000) |
| Q11. (Seven-likert points) | 5.187 (1.339) | 5.022 (1.358) | 5.226 (0.000) |
| Q12. (Seven-likert points) | 4.840 (1.332) | 4.655 (1.333) | 5.915 (0.000) |
| Q13. Number shown in % | | | $\chi^2$ (p value) |
| passing genetic changes to offspring | 1344 (62.1%) | 6101 (55.3%) | 33.992 (0.000) |
| high cost | 1304 (60.2%) | 6834 (61.9%) | 2.196 (0.138) |
| adverse medical side effects | 1491 (68.9%) | 7941 (72.0%) | 8.455 (0.004) |
| privacy | 571 (26.4%) | 3130 (28.4%) | 3.544 (0.060) |
| going against nature | 1536 (70.9%) | 6621 (60.0%) | 91.957 (0.000) |
| going against religious belief | 285 (13.2%) | 869 (7.9%) | 63.483 (0.000) |

Note: We conducted a series of independent samples t test to explore the differences in the answers between two groups.

**Table S4.** Summary of respondents' attitude to using gene therapy in children that affected by if having children. In order to examine whether having children or not has influences on participants' attitudes and cognitions to gene therapy, independent samples t tests were also employed. Results were presented in the following table.

| Questions | **Clinicians ($N$ = 2165)** | | |
|---|---|---|---|
| | Without children, mean (*SD*) | With children, mean (*SD*) | *t* (*p* value) |
| Q7 | 5.912 (1.196) | 6.073 (1.226) | -3.081 (0.002) |
| Q8 | 4.681 (1.740) | 4.765 (1.874) | -1.079 (0.281) |
| Q9 | 3.742 (1.797) | 3.794 (1.893) | -0.663 (0.507) |
| | **The public ($N$ = 11036)** | | |
| Q7 | 5.700 (1.294) | 5.783 (1.339) | -3.356 (0.001) |
| Q8 | 4.659 (1.675) | 4.761 (1.780) | -3.051 (0.002) |
| Q9 | 3.94 (1.749) | 4.28 (1.872) | -9.725 (0.000) |

Note: Children: 0 = Yes, 1 = No.

**Table S5.** Summary of standardized beta coefficients (β) from multiple linear regression analyses used for determining association of demographics and the respondents' attitudes towards gene therapy. By treating participants' demographic information (i.e., gender, age, educational levels, religion, income levels, and having friends or relatives afflicted with a genetic disease) as independent variables, and scores on each question as dependent variables, several multiple linear regression analyses were conducted in two sub-samples. Results revealed the associations between demographic variations and participants' attitudes towards the gene therapy. In each model, gender, religion and having friends/relatives afflicted with a genetic disease are dummy variables, while age, educational levels and income levels are considered to be continuous. F tests tell the significance of the whole model. When the result of F test is significant, we can move to the results of individual tests (i.e., standardized beta coefficients and the corresponding p values). The importance as well as the influential direction of each predictor can be known from the absolute values and positivity/negativity of standardized beta coefficients, respectively.

| | **Clinicians (*N* = 2165)** | | | | | | | | | | | | | | | | | | | | | | | |
|---|---|---|---|---|---|---|---|---|---|---|---|---|---|---|---|---|---|---|---|---|---|---|---|---|
| | Q1 | | Q2 | | Q3 | | Q4 | | Q5 | | Q6 | | Q7 | | Q8 | | Q9 | | Q10 | | Q11 | | Q12 | |
| | β | *p* | β | *p* | β | *p* | β | *p* | β | *p* | β | *p* | β | *p* | β | *p* | β | *p* | β | *p* | β | *p* | β | *p* |
| Gender | .030 | .160 | -.030 | .140 | .095 | .000 | .015 | .480 | .020 | .352 | .010 | .627 | .058 | .006 | .086 | .000 | -.002 | .944 | .083 | .000 | .028 | .194 | -.002 | .935 |
| Age | .049 | .027 | .020 | .363 | .058 | .010 | .018 | .428 | .034 | .132 | .043 | .057 | .018 | .419 | -.013 | .557 | .007 | .758 | .071 | .002 | .068 | .003 | .078 | .001 |
| Education | .164 | .000 | .290 | .000 | .107 | .000 | .050 | .021 | .123 | .000 | .155 | .000 | .171 | .000 | .032 | .144 | -.010 | .652 | .016 | .463 | .091 | .000 | .048 | .028 |
| Religion | -.026 | .210 | -.018 | .392 | -.028 | .193 | .019 | .372 | -.041 | .055 | -.029 | .169 | -.045 | .034 | -.004 | .870 | .006 | .785 | .013 | .547 | -.059 | .005 | -.035 | .103 |
| Income | .096 | .000 | .058 | .007 | .040 | .075 | -.018 | .430 | .043 | .054 | .082 | .000 | .067 | .002 | .069 | .002 | .018 | .427 | .038 | .096 | .049 | .030 | .039 | .084 |
| Diseased | .091 | .000 | .060 | .004 | .065 | .002 | -.030 | .163 | .062 | .004 | .046 | .034 | .075 | .000 | .029 | .185 | .005 | .803 | .026 | .226 | .090 | .000 | .049 | .024 |
| | *F* | sig | *F* | sig | *F* | sig | *F* | sig | *F* | sig | *F* | sig | *F* | sig | *F* | sig | *F* | sig | *F* | sig | *F* | sig | *F* | sig |
| | 21.095 | .000 | 38.607 | .000 | 11.999 | .000 | 1.441 | .195 | 9.824 | .000 | 15.054 | .000 | 18.566 | .000 | 5.092 | .000 | .232 | .966 | 5.690 | .000 | 11.928 | .000 | 6.171 | .000 |
| | **The public (*N* = 11036)** | | | | | | | | | | | | | | | | | | | | | | | |
| Gender | -.017 | .060 | -.087 | .000 | .047 | .000 | .020 | .038 | .005 | .586 | .008 | .400 | .021 | .022 | .070 | .000 | .038 | .000 | .042 | .000 | .015 | .108 | .012 | .214 |
| Age | .096 | .000 | -.094 | .000 | -.016 | .120 | .002 | .850 | .031 | .002 | .071 | .000 | .020 | .049 | .012 | .228 | .075 | .000 | .074 | .000 | .069 | .000 | .098 | .000 |
| Education | .172 | .000 | .125 | .000 | .033 | .001 | .027 | .006 | .049 | .000 | .053 | .000 | .097 | .000 | .007 | .501 | -.091 | .000 | -.069 | .000 | .011 | .241 | -.012 | .217 |
| Religion | .005 | .573 | .005 | .620 | -.047 | .000 | .017 | .080 | -.033 | .000 | -.034 | .000 | -.043 | .000 | .009 | .332 | .017 | .065 | -.002 | .859 | -.031 | .001 | -.003 | .713 |
| Income | .049 | .000 | .028 | .003 | .036 | .000 | .010 | .329 | .032 | .001 | .042 | .000 | .019 | .052 | .029 | .003 | .013 | .165 | .017 | .087 | .035 | .000 | .020 | .039 |

| | | | | | | | | | | | | | | | | | | | | | | | | |
|---|---|---|---|---|---|---|---|---|---|---|---|---|---|---|---|---|---|---|---|---|---|---|---|---|
| Diseased | .114 | .000 | .041 | .000 | .071 | .000 | -.003 | .761 | .104 | .000 | .110 | .000 | .124 | .000 | .048 | .000 | -.015 | .114 | .020 | .042 | .087 | .000 | .052 | .000 |
| | *F* | sig | *F* | sig | *F* | sig | *F* | sig | *F* | sig | *F* | sig | *F* | sig | *F* | sig | *F* | sig | *F* | sig | *F* | sig | *F* | sig |
| | 120.039 | .000 | 69.497 | .000 | 23.304 | .000 | 2.731 | .012 | 35.389 | .000 | 52.272 | .000 | 58.397 | .000 | 16.940 | .000 | 33.567 | .000 | 27.506 | .000 | 34.109 | .000 | 29.947 | .000 |

Note: Standardized beta coefficients are reported. Income levels: Mean = 3.00, SD = 0.742 (clinician); Mean = 2.89, SD = 0.740 (the public).
Gender: 0 = Male, 1 = Female; Religion: 0 = Religious, 1 = Not religious; Having friends or relatives afflicted with a genetic disease: 0 = Yes, 1 = No.

**SUPPLEMENTAL FIGURES**

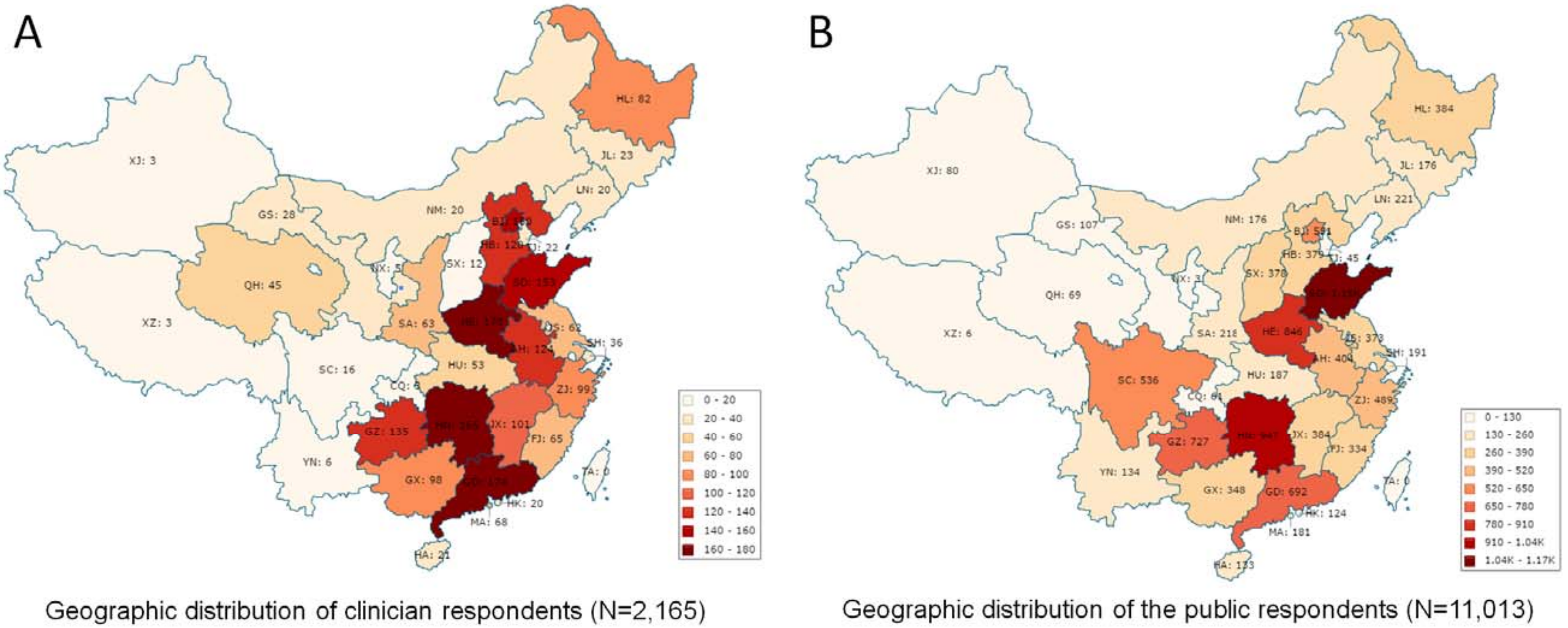


**Figure S1.** Geographic distributions of valid respondents across China. (A) Geographic distribution of clinician respondents (N=2,165) (B) Geographic distribution of the public respondents (N=11,013). Respondents from both groups were proportionally distributed across China based on the population distribution in each province.

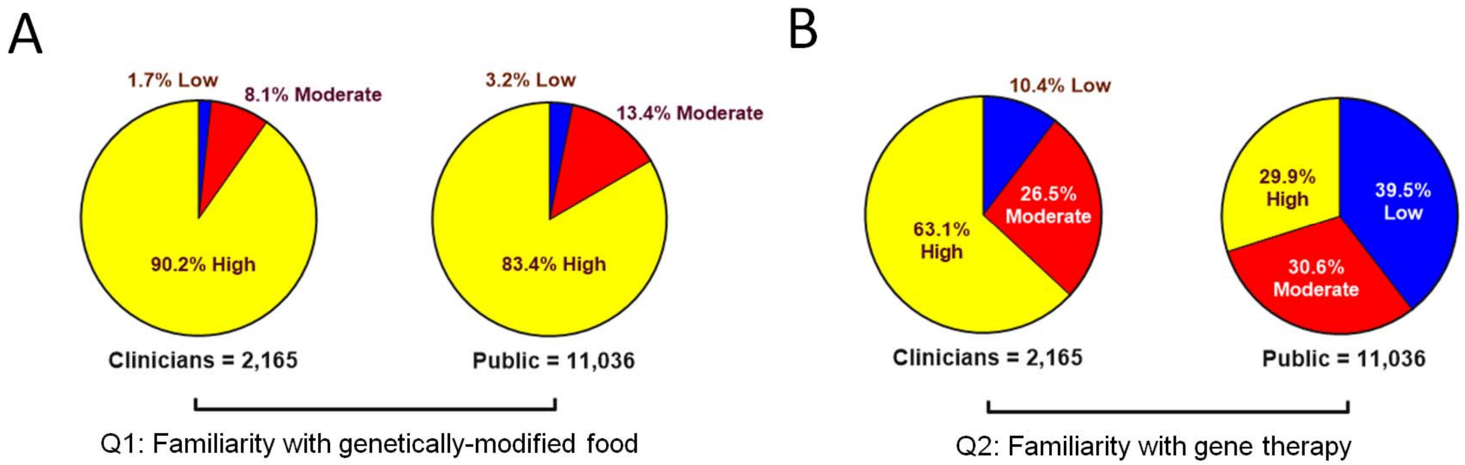


**Figure S2.** Proportion of respondents being familiar with genetically-modified food and gene therapy. (A) Proportion of respondents being familiar with genetically-modified food. Both the clinicians and public respondents showed the significantly high familiarity with GM food (90.2 % and 83.4%, respectively). (B) Proportion of respondents being familiar with gene therapy. The majority of clinicians (63.1%) had the remarkably higher familiarity with gene therapy than the public (29.9%). High represents the respondents were highly familiar with the term. Low represents the respondents were less familiar with the term. Moderate familiarity is in the between.

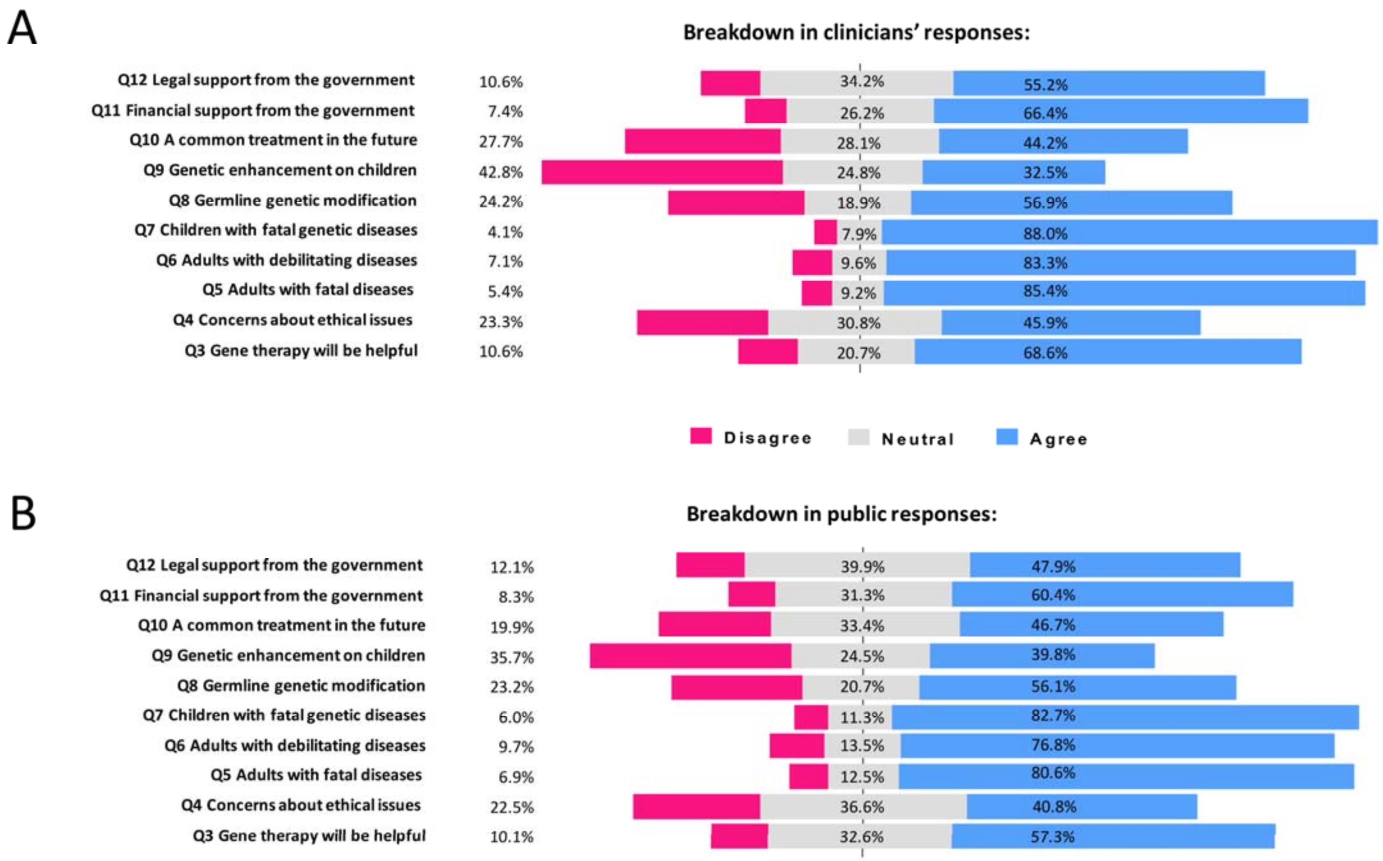


**Figure S3.** The proportion of clinicians' and public attitudes toward gene therapy. Responders who selected strongly disagree, disagree, mildly disagree for the individual question were merged to the population with the attitude of a disagreement. Responders who selected mildly agree, agree, strongly agree for the individual question were merged to the population with the attitude of an agreement.

**SUPPLEMENTAL REFERENCES**


1. Wang, YX, and Li, YT (2016). AME survey-003 A2: on the attractiveness of an medicine career in current China with a survey of 7,508 medical professionals and 443 non-medical professionals. *Quantitative imaging in medicine and surgery* **6**: 84-102.
2. McCaughey, T, Sanfilippo, PG, Gooden, GE, Budden, DM, Fan, L, Fenwick, E, *et al.* (2016). A Global Social Media Survey of Attitudes to Human Genome Editing. *Cell stem cell* **18**: 569-572.
3. Blendon, RJ, Gorski, MT, and Benson, JM (2016). The Public and the Gene-Editing Revolution. *The New England journal of medicine* **374**: 1406-1411.
4. Xiang, L, Xiao, L, Gou, Z, Li, M, Zhang, W, Wang, H, *et al.* (2015). Survey of Attitudes and Ethical Concerns Related to Gene Therapy Among Medical Students and Postgraduates in China. *Human gene therapy* **26**: 841-849.
